# Content validity of the Dutch Rheumatoid Arthritis Impact of Disease (RAID) score: results of focus group discussions in established rheumatoid arthritis patients and comparison with the International Classification of Functioning, Disability and Health core set for rheumatoid arthritis

**DOI:** 10.1186/s13075-015-0911-z

**Published:** 2016-01-22

**Authors:** Marieke M. ter Wee, Lilian H. van Tuyl, Birgit S. Blomjous, Willem F. Lems, Maarten Boers, Caroline B. Terwee

**Affiliations:** Amsterdam Rheumatology and Immunology Center, VU University Medical Center and Reade, Amsterdam, The Netherlands; Department of Epidemiology and Biostatistics, VU University Medical Center, Amsterdam, The Netherlands; EMGO Institute of Health and Care Research, VU University Medical Center, Amsterdam, The Netherlands

**Keywords:** Rheumatoid arthritis, RAID score, WHO ICF, qualitative study, COSMIN standard

## Abstract

**Background:**

The Rheumatoid Arthritis Impact of Disease (RAID) score was developed as a European League Against Rheumatism initiative to obtain a patient reported outcome score for clinical trials in patients with rheumatoid arthritis (RA), based on patients’ perception of the impact of the disease on several domains of health. The objective of this study was to assess the content validity of this score in Dutch RA patients.

**Methods:**

During three focus group discussions (n = 23), patients with RA reflected on comprehensiveness of the RAID to measure impact of RA on their life, relevance of the RAID domains and formulation of questions. Also, the domains of the RAID score were compared to the comprehensive International Classification of Functioning, Disability and Health core set for RA.

**Results:**

Patients confirmed that RA had impact on five domains already incorporated in the RAID score: emotional well-being, pain, performing daily activities, fatigue and coping. There was variation in interpretation of some of the items of the RAID score, suggesting problems in comprehension. Patients indicated that the domains work, relationships with others (such as family and friends) and spare time/hobbies were missed in the RAID and could be added to obtain a more ‘complete’ picture of the impact of the disease.

**Conclusion:**

The RAID score has fairly good content validity. If confirmed as important in other patient groups, items in the above mentioned areas should be considered in a future upgrade.

**Electronic supplementary material:**

The online version of this article (doi:10.1186/s13075-015-0911-z) contains supplementary material, which is available to authorized users.

## Background

Rheumatoid arthritis (RA) is a chronic inflammatory joint disease that strongly affects quality of life [1–3]. The core set of outcome measures for RA clinical trials includes three patient-reported outcomes (PROs): pain, physical functioning and patient global assessment of disease activity [4]. In addition, the Outcome Measures in Rheumatology (OMERACT) group, an independent initiative of international health professionals and patient experts interested in outcome measures in rheumatology, has recommended that fatigue should also be measured in all RA clinical trials; furthermore, sleep quality and ability to work have both been mentioned as important outcomes and targeted for further instrument development [5–7]. Several questionnaires now exist to assess these PROs separately [8–10].

Recently the Rheumatoid Arthritis Impact of Disease (RAID) score was developed as an initiative of the European League Against Rheumatism (EULAR) to combine the most important PROs in one measure [11]. This patient-centered response index for clinical trials in RA was to replace the use of different questionnaires per outcome without missing important information, and to assess changes in outcome over time [12]. In 2011, the construct validity, reliability and sensitivity to change of the RAID score were documented in an international study. A strong correlation was found with other disease activity measures (e.g., patient global assessment visual analogue scale (R = 0.76)), as well as a high reliability (intraclass correlation coefficient: 0.90) and a good sensitivity to change (standardized response mean: 0.9) [13]. As the domains of the RAID were initially identified by a relatively small group of patient partners (n = 10), we decided to assess the content validity of the RAID questionnaire as well as comprehensibility before implementation of the RAID score in the Netherlands.

## Materials and methods

### Development of the RAID score

The development of the RAID score has been described elsewhere [12]. In brief, a priori the developers decided to create an instrument with seven domains. In the first phase, domains were initially elaborated through a ‘focus group’-type meeting where 10 patients (one patient per country, ten countries participated) identified 17 health domains on which RA had impact, based on patients’ personal experiences. To reduce the list of domains, 100 new patients ranked these domains in order of decreasing impact of RA on their lives.

In phase 2, items to measure the candidate domains were identified. One or several items, instruments or whole questionnaires were selected for each domain. When no validated instruments were available, a numerical rating scale was formulated by the group and validated with the 10 patients with arthritis who participated in the first phase. In the third phase, relative weights of the candidate domains were obtained, in which the second part of phase 1 was repeated with 500 patients. In the last phase, the generalizability of the preliminary RAID was assessed by comparing ranks of importance of chosen domains across countries (using data from phase 1), and by analyzing weights across disease and demographic characteristics. The final RAID score covers seven health domains: pain, problems with daily functioning, fatigue, sleep, physical well-being, emotional well-being and coping with RA, each represented by a numerical rating scale ranging from 0 to 10 [12]. The complete questionnaire and related calculation rules can be found in Additional file 1.

The translation of the questionnaire into Dutch was performed by the RAID developers. Two independent researchers (with Dutch as their mother tongue) translated the questionnaire into Dutch. After reaching consensus, back-translation was performed by the same two researchers. A multidisciplinary consensus meeting was held in which the original version and the back-translation were compared and in the last step the final version was pre-tested on five patients [12].

### Current study: content validity

Content validity as defined by the Consensus-based Standards for the selection of health Measurement Instruments (COSMIN) group addresses the question: are all items included in the RAID score an adequate reflection of the construct to be measured? Content validity is often considered one of the most important measurement properties and therefore addressed first, before looking at other measurement properties such as construct validity. Construct validity is defined as the degree to which the scores of the RAID score are consistent with hypotheses that were made in advance (e.g., about relation of scores to scores of other instruments) based on the assumption that the RAID score validly measures the construct to be measured [14].

This study used a part of the COSMIN checklist to evaluate the content validity of the RAID [15, 16]. This checklist was developed to evaluate the methodological quality of studies on measurement properties [15]. The items in box D of the COSMIN checklist were slightly adapted to evaluate the quality of the instrument. Evidence was collected to answer the following four questions, based on box D of the COSMIN checklist, as evidence for content validity: 1) Do all items refer to relevant aspects of the construct to be measured? 2) Are all items relevant for the study population? 3) Are all items relevant for the purpose of the measurement instrument? And 4) do all items together comprehensively reflect the construct to be measured?

### Focus group discussions

To gain insight into the domains of health upon which RA impacts, patients were asked to participate in focus group discussions (FGDs). A FGD is a form of qualitative research that retrieves data on patients’ experiences, opinions, perspectives and assumptions [17]. In a FGD patients are engaged to interact with each other. In this study, patients were asked to describe the impact of RA on their life (question 2 of box B), and whether they felt that the RAID score covered all items that should be measured to assess the impact of RA on their life (question 4 of box B). We were also interested to assess the comprehensibility of the RAID. Therefore, in the second part of the FGD, patients were asked if they felt that the RAID questions were understandable and clearly defined (see Table 1 for the structure of the focus group discussions).

**Table 1:** Basic structure of the group focus discussions

1. Could you introduce yourself and tell us how long ago rheumatoid arthritis (RA) was diagnosed?
2. If you think of all aspects of your life RA had influence on, what is the first thing that pops up in your mind?
Introduction of RAID score by LHvT to the participants:
Recently, a group of people in Europe developed a questionnaire that measures the impact of RA on the patient's life on various health topics. Before a questionnaire can be used in daily practice or research, it must first be examined whether the questionnaire works properly and is reliable. That is why we are here today. The purpose of this meeting is to investigate whether you think that this questionnaire is complete, or if you miss domains/subjects in this questionnaire. In response to a question I will ask you, you can discuss and debate your answer with each other. In the meantime I will keep an eye on the equal input of everyone. The entire meeting is recorded on tape that we will analyze later. After about 45 minutes we will have a break of 15 minutes. The persons who developed the list found that the questionnaire had to remain short. Therefore there are seven topics included in this questionnaire, as you can see on the paper version we just provided you with. The topics include pain, problems with daily functioning, fatigue, sleep, physical well-being, emotional well-being and handling your disease.
3. What do you think of these topics?
4. Are these seven topics the most relevant to assess the impact of RA on your life?
Second introduction of LHvT after the break:
In the second part of this meeting, we would like to evaluate the questions and how they are formulated to see if everything is understandable and clear to you.
5. Evaluation of all questions separately by asking if all questions were clearly defined and understandable.
6. Today, I wanted to gain insight into all the domains in which RA had impact on in your life. Have I forgotten to ask you something that relates to this subject? Is there something you would like to say if you did not have the opportunity during the discussions? Would you like to add something to the previous discussions?

#### Study sample focus group discussions

To gather as much diversified opinion from patients as possible, patients with diagnosed RA (≥18 years and not suffering from multiple co-morbidities) were recruited through two patient associations in Amsterdam and surroundings to participate in one of three planned FGDs (maximum 10 patients per group). These patient associations distributed an invitation letter to their members.

#### Data collection

All questions were open-ended [18]. Table 1 shows the FGD structure used. Data (on the domains mentioned by the patients) were gathered until saturation was reached. With data saturation, focus group interviews were performed until no new categories, themes or explanations emerged from the patients [19]. Patients were informed that the FGDs were moderated (LHvT), taped and anonymously transcribed (BSB) by researchers not involved in the clinical management of any of the patients. Under Dutch law, this study does not need approval from an Ethical Review Board. However, all patients gave oral informed consent at the start of the group sessions on the use of their provided responses, which was recorded.

#### Data analyses

To thoroughly investigate all expressions of the patients, the focus group moderator asked in-depth questions such as: ‘In what way do you mean that?’ or ‘Could you provide us with an example?’ The data were analyzed using the interpretative phenomenological analyses method that assesses content as well as the underlying cognitive and emotional concepts [20, 21]. A bottom-up approach is applied to avoid prior assumptions and minimize bias. Two researchers experienced in qualitative research (MMtW and LHvT) systematically and independently analyzed the transcripts to identify relevant themes, and subsequently agreed to the same set of major themes during a consensus meeting. The researchers also discussed whether all RAID items potentially could show change when the disease improves or deteriorates (question 3 of COSMIN box D).

### World Health Organization International Classification of Functioning, Disability and Health core set for RA comparison

In order to answer question 1 of COSMIN box D, the domains in the RAID score were compared to the domains in the World Health Organization (WHO) comprehensive core set of International Classification of Functioning, Disability and Health (ICF) for RA to see whether all items refer to relevant aspects of the construct to be measured (see Additional file 2) [22]. The ICF makes it possible to assess not only the medical aspects of the patients’ disease, but also to take into account all aspects of their life such as participation and environment [23]. ICF core sets, such as the ICF core set for RA, have been developed to describe a patient’s level of functioning specifically for one disease as only the relevant categories for this disease are listed in the core set [24]. Previously, several studies assessed the validity of the ICF core set for RA. In one of these studies, patients almost entirely confirmed the domains included in the core set in several focus group meetings [25].

## Results

### Focus group discussions

The two patient organizations sent the invitation letter to all their members (approximately 600). A total of 23 patients were willing to participate in the focus groups, but five subsequently declined, mostly for lack of time. The remaining 18 patients were split equally among three FGDs. Mean age was 60 years (standard deviation 17 years), median disease duration 14 years (range 2–50 years), and 89 % of patients were women. Approximately 50 % of the patients had a high educational level (>17 years) and 17 % a low level (≤12 years). Most patients were either married or living together (83 %). Regarding work status, 28 % performed paid work (17 % worked ≥32 hours per week), 28 % were retired and 39 % were fully work disabled. The results of the interviews are presented using summaries and quotes. During the third focus group interview, no new categories, themes or explanations emerged from the patients (data saturation). Therefore, no new patients had to be recruited.

Performing or maintaining their job was one of the first things patients mentioned in all three FGDs. Most patients lost their job due to their disease. This made patients uncertain about their financial situation and it affected their self-confidence, both leading to feelings of stress and increasing their perceived disease activity. Patients who were still working noticed that pain and fatigue decreased their work productivity. Quotes 1–4 in Table 2 concern this topic.

**Table 2:** Quotes of participants on work and coping with the disease

Quote number	Quotes
Performing or maintaining a job	
1	P1.5: Almost 4 years ago I got acute and complete RA manifestations in almost all of my limbs, symmetrical. Everything fell apart. I use the normal ‘MTX misery’ and I’m greatly improved. I have a very positive look on everything. But my whole worldwide career was over and out in one stroke.
2	P1.7: You will not be cured. You can say goodbye to that. But you can adapt to what you ARE able to do. You don’t have to look at what you can’t do.
	P1.2: That kept me at work for 20 years.
	P1.7: They threw me out of my job because I visited the doctor too often.
3	P2.3: Yes, at a certain point I had to decide that I would step back from my company because I physically couldn’t handle it anymore. But I kept at it long enough to get the business on its feet.
	P2.2: And that’s pretty difficult, when you’ve started it yourself, such a business.
	P2.3: You bet.
	P2.2: So you’re completely out of it?
	P2.3: Yes, in the end I was declared work disabled, but the company is still running and I’m the nicest volunteer there.
4	P3.4: I’ve had arthritis for the last 18 years and especially the last 5 years it has had enormous impact. It went pretty well for quite a long time. The first year was really bad, I was on sick leave the whole year. Then, after starting with medication, actually, I started building it up again slowly, and up to 5 years ago it was sort of stable. And now it’s so bad, especially the last half year, that sometimes I think I’m just going to let everything drop and quit. It might not look like that today, but that’s the way it is…
	P3.2: It varies from day to day.
	P3.4: Yes, yes, so actually I’ve now been declared fully disabled for work.
Coping with the disease	
5	P2.1: The limitations are a constant search for what your limits are, I find that very difficult. And I’m also finding out that even after all those years, I don’t really know the balance, because I don’t know. The moment I feel up to it, I go all the way and then a few days later you get a relapse, so that is bothersome. Walking is hard, you have pain, fatigue. So there is always a sort of constant considerations what to do. Sometimes you get a flare if you’ve gone too far. So you’re constantly making do and I find that very hard.
6	P2.5: Sometimes it’s really weird, because then you want to pick up a pen and you can’t for some reason… your hand locks down and you can’t get the pen into your hand. And sometimes I can suddenly have a day where my leg really hurts, but not the joints, just the muscle, and it lasts for a day and then it is gone and I really drag my feet and stumble around for a day and the next day it is gone.
7	P2.3: And then somebody says: are you coming sailing this weekend. And I say: yes! And then the first day goes well, and the second day slightly less, and then you get home and the next three days you’re sort of knocked out a little. And, so, I really have to make a positive choice. I just feel like to be out on the water, too bad. Then the week after I’m washed out, sort of. You should stop when it is still going really well.
	P2.1: And that’s strange!
	P2.3: And I don’t want to…
	P2.1: No, you don’t.
8	P3.2: You have to be very disciplined in the way you allocate your energy.
	P3.6: Yes, yes.
	P3.2: And you know that’s really a long process.
	P3.5: Yes. Safeguarding your energy level, that I find the most difficult.
	P3.6: Right. I’d forgotten about that. Energy is finished, you can, you want a lot, but at a certain moment you just can’t anymore.

Information on participants: P1.2 (F, 57 years); P1.5 (M, 64 years); P1.7 (F, 65 years); P2.1 (F, 41 years); P2.2 (F, 65 years); P2.3 (F, 58 years); P2.5 (F, 43 years); P3.2 (F, 57 years); P3.4 (F, 56 years); P3.5 (F, 46 years); P3.6 (F, 63 years). *F* female, *M* male, *MTX* methotrexate, *RA* rheumatoid arthritis

A second major issue for patients was the difficulty in coping with their disease. RA can fluctuate with regard to disease activity during the day, making it possible to perform an activity at one point in time but impossible at the next. They also mentioned they experienced difficulties with coping and had to make other choices due to their disease. Patients indicated that this was because it felt as if their body would ‘abandon’ them while performing an activity. Quotes 5–8 in Table 2 concern this topic.

The third domain that patients discussed was the influence of their disease on relationships with others, such as partners, children, and friends. Patients experienced a lack of understanding and consideration on days that their disease was very active; this was attributed to the relatively ‘invisible’ nature of RA as a disease as, for example, compared to a broken leg. Due to the disease, the distribution of roles changed. For example, partners would have to perform more household tasks than they had to do before their partner was diagnosed with RA; or patients were not able to engage in activities with friends as they used to. Therefore, irritations, tension and stress could occur in the relationship with others, sometimes leading to losing a partner or friend. Quotes 9–11 in Table 3 concern this topic.

**Table 3:** Quotes of participants on relationships with others and performing activities in daily life and leisure time

Quote number	Quotes
Relationships with others (partners, children and friends)	
9	P1.2: You’re in a relationship in a certain way, and if that changes totally, then you can’t, well, blame either one that it doesn’t work anymore. Hey, you lose your job, you’re suddenly sitting at home, you need help and he (patient’s partner) certainly didn’t have that patience. I mean, before I was in the car, there was a period that I was really very stiff and my leg would still be outside and he would step on the gas. He was already driving off! So when it was over I felt like, I don’t have to keep up the pretense. In my marriage, especially the last year, it was very often simply overstretching too much. Because, you know, you want to keep up, be part of it. And be appreciated in that way, so you accept the situation. I couldn’t then, but I can now.
10	P2.1: Even my partner has something …, he understands, but then again he doesn’t.
	P2.4: That sounds very familiar.
	P2.1: Especially in times when it’s heavy for me, he says: ‘come on, just this little thing’. But then I think: ‘right now you have to leave me be’. And that, that’s so difficult, because if we can’t understand it ourselves, then the other can’t either.
	P2.2: No, because in fact the slap on the wrist always comes too late.
	P2.5: You never get a warning signal saying: now you should stop.
11	P3.2: I’ve always been a busybody who never stopped going but at a certain moment, I had a friend, and really, my best friend, she just didn’t have a lot of energy intrinsically, and she said at one point, when I became ill, she just didn’t get it. And she said at a certain point, well then I actually got very mad at her, she says: ‘that disease just doesn’t suit you, I don’t understand that you…’ And then I said, ‘well, I think the disease chooses…’
	P3.5: Whom does it suit then?
	P3.2: …randomly selects someone. How can you say the disease doesn’t suit me? ‘Yes but you always had so much energy’. I say: ‘But I didn’t ask for it, did I?’ Also, she’s the one of all my friends who is the least realistic in coping with my disease, she’s still surprised when I’m not able to do something.
Performing activities in daily life and leisure time	
12	P1.4: Yes, you can’t dress yourself anymore. You can’t wipe your bum anymore. You feel, in that moment, you feel a bit powerless. A bit. Look I wasn’t working anymore, but then you really feel switched off.
	P1.7: Dependent…
	P1.4: Dependent. I mean, if you can’t even slice your own bread…
13	P2.5: When I’m walking somewhere… If I walk for a really long time, or if I’m on a shopping spree with the girls, then really after an hour, then I have to sit down for a bit.
14	P3.4: I’m a big fan of the Antilles. Usually I go with friends, but this winter I’m alone because nobody can get days off. Then I think: oh, should I be doing this? Because then I’ll be there and not able to do anything. So those are, those are actually, small things but…
	P3.6: Limitations you have in your life.
	P3.2: But don’t you, when you’re there, don’t you have a lot less complaints, because, I swear I really…
	P3.5: Warmth
	P3.2: …That really has an impact on my body.
	P3.4: True, but the flight already takes you a few days to get over.
	P3.2: And sitting still all that time.
	P3.4: And 6 hours of time difference.
	P3.6: Yes, that’s what I mean, I can’t do that. That’s really difficult, traveling is.
	P3.4: Yes, so before you’ve slightly…
	P3.5: Simply recovered from.
	P3.4: And then the fear of, suppose I get something there? Then I think: oh, should I be doing this?

Information on participants: P1.2 (F, 57 years); P1.4 (F, 65 years); P1.7 (F, 65 years); P2.1 (F, 41 years); P2.2 (F, 65 years); P2.4 (F, 79 years); P2.5 (F, 43 years); P3.2 (F, 57 years); P3.4 (F, 56 years); P3.5 (F, 46 years); P3.6 (F, 63 years). *F* female, *M* male

A fourth domain that was influenced by RA was performing activities in daily life and in spare time. Regarding routine daily activities, patients reported having difficulty getting dressed, taking care of their children, cooking, going to the toilet and performing household activities. Regarding leisure-time activities, patients mentioned that it became difficult to perform their sports, to go on vacation, or perform a hobby such as painting. Quotes 12–14 in Table 3 concern this topic.

Three other domains that patients mentioned that impacts their life were having pain, being fatigued and being emotional. Having pain and being fatigued restricts functioning in all aspects of life, but having the disease also results in emotional fluctuations. For example, patients experience anger towards their disease as it sometimes limits their functioning. Or they have the urge to cry due to pain, or get frustrated due to the inability to perform a task they were able to perform the day before. Quotes 15–20 in Table 4 concern this topic.

**Table 4:** Quotes of participants on pain, fatigue and emotions

Quote number	Quotes
Pain	
15	P1.3: That there are things you can’t do, well, you can solve that in another way. But the pain I think is the worst.
	P1.4: It’s so tiring, isn’t it?
	P1.6: Yes terrible.
Fatigue	
16	P3.5: The problem is simply that I feel very insecure about fatigue. The fatigue that, that I…
	P3.6: Fatigue, yes, that’s right.
	P3.5: … notice all the time, I find it very bothersome to function around noon, when I’ve worked I simply have to sleep for an hour.
Emotions	
17	P1.1: The first thing that popped up when I was asked to participate in these interviews was a kind of old rage from 15 years ago that it happened to me.
18	P1.2: This last period so many things are happening one after the other, eh, because of this medication, all kinds of side effects, that I’m really angry and also a bit desperate.
19	P2.3: There was a moment I had the feeling of why does this have to happen to me?
20	P2.5: I notice that at times when I’m really tired, I can be really grumpy with those around me.

Information on participants: P1.1 (F, 65 years); P1.2 (F, 57 years); P1.3 (F, 48 years); P1.4 (F, 65 years); P1.6 (F, 72 years); P2.3 (F, 58 years); P2.5 (F, 43 years); P3.5 (F, 46 years); P3.6 (F, 63 years). *F* female, *M* male

From the seven domains in the RAID score, five were indicated as being relevant for the study population: a) coping with the disease; b) functional disability assessment (activities performed in daily life); c) pain; d) fatigue, and e) emotional well-being. The domains sleep and physical well-being were briefly or not at all mentioned in the FGDs. The domains work, relationships with third parties and leisure time activities are considered important and are missed in the RAID score by the patients.

During their consensus meeting, the two researchers discussed whether the RAID score can be used as an evaluative instrument, i.e., to assess change over time-for example, the effect of drugs on the impact of the disease [12]. They considered all items potentially changeable, and therefore the whole RAID score was considered relevant for an evaluative purpose.

#### Comprehensibility

In general, the numerical rating scales may cause difficulties in interpretation. In the Netherlands such questions are often answered by comparing them with school grades (a grade 10 is outstanding and grade 1 is very poor; however, in practice only the grades 4–9 are used). In other words, the full range of the scale may not be used, and patients need to reverse the anchors, as low rather than high scores in the scales reflect the preferred condition, which was perceived as counterintuitive.

Although the patients found the questions on daily physical functioning, fatigue, sleep and emotional well-being clearly defined, they also mentioned that it was difficult to confidently ascribe fatigue or sleeping problems to RA, as these are also influenced by other circumstances. The questions about daily physical functioning and emotional well-being were interpreted by the patients as having problems with performing household activities, and coping with their disease in the past week, respectively. It might be more clear when concrete descriptions of activities are provided instead of broad concepts, e.g., by referring to hobbies and work instead of referring to daily physical functioning.

Patients indicated that the questions on pain, physical well-being and coping with RA were difficult to understand, and not clearly formulated. Patients described pain to be dependent on the type of activities that are performed, and influenced by different (environmental) circumstances. Therefore, pain perception can change every hour making it difficult to give an average rating over 1 week. They stated that time frames other than 1 week (for example 1 day) might be more appropriate to use. Concerning the question on coping, patients indicated they felt this question covered the same domain as the question on emotional well-being. They were unclear whether the subject of this question concerns coping with RA emotionally, in daily functioning or in general. For coping, a time period of 3 months was found to be more appropriate than 1 week.

Concerning the question on physical well-being, it seems that translation errors have occurred. For example, in the Dutch version a sentence has been added to the question on physical well-being: ‘considering your physical well-being (*apart from pain, inflammation and fatigue*)’, which is not mentioned in the English version (see Additional file 1). Patients indicated that it is impossible to set aside the pain, inflammation and tiredness in answering this question, as these items influence the functioning of their body and therefore their physical well-being.

### WHO ICF core set for RA comparison

The WHO ICF core set for RA comprises 96 categories, divided over the components ‘Body structures’, ‘Body functions’, ‘Activities and participation’, and ‘Environmental factors’ (see Additional file 2) [22]. Table 5 shows the results of the comparison of the components of the ICF core set and the domains as measured in the RAID score. Most of the domains are formulated as broad concepts and cover several items of the ICF core set. Fatigue and coping are not mentioned in the ICF core set. These could be linked to third level categories ‘b1300 Energy level’ and ‘b4552 fatigability’ of the entire WHO ICF framework, but fatigue is not specifically and explicitly mentioned in the core set [24–27]. The component ‘Environmental factors’ of the core set are not covered in the RAID score, as well as the levels d660–d920 of the component ‘Activities and participation’. Concerning pain the ICF core set contains far more specified pain categories, at the third level specified per body part, which are not addressed by the RAID. Finally, all components of ‘Body structures’ (e.g., structure of lower or upper extremity) and ‘Body functions’ (e.g., mobility of joint functions, sensations related to muscles and movement functions) are also not covered in the RAID. However, such items are not expected to be measured in a PRO measure.

**Table 5:** Comparison of the RAID score with the WHO ICF core set for RA

WHO ICF core set for RA	RAID score
Body functions	1. Pain
	2. Sleep
	3. Physical well-being
	4. Emotional well-being
Body structures	Physical well-being
Activities and participation	Functional disability assessment
Environmental factors	Coping
	Fatigue

*ICF* International Classification of Functioning, Disability and Health, *RA* rheumatoid arthritis, *RAID* Rheumatoid Arthritis Impact of Disease, *WHO* World Health Organization

In conclusion, five out of the seven items from the RAID score refer to three domains of the WHO ICF core set. RAID adds two domains not covered by the WHO ICF, and omits four, of which two are outside the scope of a PRO measure.

## Discussion

Taking into account the impact of RA disease activity on the daily life of patients (the aim of the RAID score) is of high importance to rheumatology clinical practice. Our study confirms the importance of five domains of the RAID (coping with the disease; functional disability assessment (activities performed in daily life); pain; fatigue, and emotional well-being). Our patients also noted the omission of questions on (paid) work, relationships with others, and activities performed in spare time.

In the comparison with the ICF core set, all categories of the components ‘Body functions’ and ‘Body structures’ were covered in the domains sleep, physical well-being, pain and emotional well-being. Approximately 75 % of the categories in the component ‘Activities and participation’ and none of the categories in the component ‘Environmental factors’ were covered by the RAID score. On the other hand, the domains fatigue and coping which are known to be of high importance to RA patients are not incorporated in the ICF core set for RA, pointing to content validity problems with the ICF core set [7, 27].

Patients also pointed out some issues with comprehension. This might partly be explained by translation errors, but also due to different interpretation of the numerical rating scales, the large time scale of 3 months to refer to, and that the questions do not provide examples to think about. The Dutch version of the RAID needs to be modified to reflect the English version, and during the development of this paper we learned this has indeed been done in 2015 (see online: http://www.eular.org/tools_products_.cfm).

The strength of our study lies in the three FGDs performed with RA patients, as this method is an effective way to discover people’s ideas, feelings and needs about a subject, in this case the impact of RA on several domains of their life [17]. Another advantage of FGDs is that they present a more usual setting in comparison to individual interviews as patients are influenced by and influencing others, just as in daily life, providing the opportunity for discussion and consensus [17]. Our patients were not provided with literature or the RAID score in advance, so they had no prior knowledge on this subject. The ten patients who participated in the original development of the RAID score are all OMERACT patient research partners. These patients have more knowledge on research than average patients. Although 50 % of our patients were also highly educated, the rest had moderate or low educational levels. Therefore we think our population, albeit small, better represents the entire RA population. We did find it striking to see that the domains sleep and physical well-being were briefly or not at all mentioned by the patients participating in our study, in comparison to the patient research partners from OMERACT who are all also patients who did mention these items. We do not have an explanation for this difference in outcome.

A limitation of this study concerns the recruitment of patients which could have biased the results. As patient organizations distributed the invitation letter, we do not know the exact number of invited patients. It is likely that patients who have noticed that their disease altered their life situation were more likely to participate in the FGDs compared to patients who did not notice a major impact of RA on their lives. Also our patients all had established RA, perhaps limiting generalizability. We did not retrieve data of other comorbidities that patients might have. Other comorbidities might also have impact on a patient’s life. Secondly, the transcript analysts were familiar with the domains of the RAID score. Therefore possible bias towards these domains might have occurred. However, as saturation was reached after the third FGD and patients were only asked about their own experiences, we think this potential bias has a minimum impact. Finally, the domains noted by Dutch patients might be weighed differently by patients in other countries.

Without question, the RAID is a major advance in the assessment of impact of RA disease activity on everyday life, and is useful in its current form. But at its presentation, we noted that some of the domains assessed by the RAID are already in the RA core set, and cautioned against double counting [28]. In this study we suggest other domains that might be added to enhance content validity from the patient perspective. Several studies have pointed out that domains such as (paid) work, relationships with others, and activities performed in spare time are important and have impact on a patient’s ‘identity’ and quality of life [5–7, 28, 29]. A future upgrade of the tool should take these findings into consideration.

## Conclusion

In conclusion, the Dutch version of the RAID score has fairly good content validity. More research is needed to confirm whether the domains (paid) work, relationships with others and activities performed in spare time are important in other patient groups. If so, these should be considered in a future upgrade.

### Ethics statement

Under Dutch law, this study does not need approval from an Ethical Review Board. However, all patients gave oral informed consent at the start of the group sessions on the use of their provided responses, which was recorded.

### Consent statement

All authors consent with the content of this article.

## Additional files

Additional file 1:
**RAID score and calculating rules.** The RAID score is explained, all questions are shown and the calculation rule to retrieve the sum score is explained. (DOCX 22 kb) Additional file 2:
**WHO ICF score set for RA. The total WHO ICF core set for RA is shown.** All categories are described in detail. (DOCX 21 kb)

## Abbreviations

COSMIN: Consensus-based Standards for the selection of health Measurement Instruments
FGD: Focus group discussion
ICF: International Classification of Functioning, Disability and Health
OMERACT: Outcome Measures in Rheumatology
PRO: Patient-reported outcome
RA: Rheumatoid arthritis
RAID: Rheumatoid Arthritis Impact of Disease
WHO: World Health Organization
