# Supplementary material for: Content validity of the Dutch Rheumatoid Arthritis Impact of Disease (RAID) score: results of focus group discussions in established rheumatoid arthritis patients and comparison with the International Classification of Functioning, Disability and Health core set for rheumatoid arthritis
Source: Arthritis Res Ther. 2016 Jan 22;18:22. doi: 10.1186/s13075-015-0911-z (PMC4722755; doi:10.1186/s13075-015-0911-z)
Supplement: Additional file 1: — RAID score and calculating rules. The RAID score is explained, all questions are shown and the calculation rule to retrieve the sum score is explained. (DOCX 22 kb) [file 13075_2015_911_MOESM1_ESM.docx]

**APPENDIX 1** RAID score and calculating rules

1. **Pain**Circle the number that best describes the pain you felt due to your rheumatoid arthritis during the last week:

| None | 0 | 1 | 2 | 3 | 4 | 5 | 6 | 7 | 8 | 9 | 10 | Extreme |
| --- | --- | --- | --- | --- | --- | --- | --- | --- | --- | --- | --- | --- |

1. **Functional disability assessment**
   Circle the number that best describes the difficulty you had in doing daily physical activities due to your rheumatoid arthritis during the last week.

| No difficulty | 0 | 1 | 2 | 3 | 4 | 5 | 6 | 7 | 8 | 9 | 10 | Extreme difficulty |
| --- | --- | --- | --- | --- | --- | --- | --- | --- | --- | --- | --- | --- |

1. **Fatigue**Circle the number that best describes how much fatigue you felt due to your rheumatoid arthritis during the last week.

| No fatigue | 0 | 1 | 2 | 3 | 4 | 5 | 6 | 7 | 8 | 9 | 10 | Totally exhausted |
| --- | --- | --- | --- | --- | --- | --- | --- | --- | --- | --- | --- | --- |

1. **Sleep**Circle the number that best describes the sleep difficulties (i.e., resting at night) you felt due to your rheumatoid arthritis during the last week.

| No difficulty | 0 | 1 | 2 | 3 | 4 | 5 | 6 | 7 | 8 | 9 | 10 | Extreme difficulty |
| --- | --- | --- | --- | --- | --- | --- | --- | --- | --- | --- | --- | --- |

1. **Physical well-being**
   Considering your arthritis overall, how would you rate your level of physical well-being during the past week? Circle the number that best describes your level of physical well-being.

| Very good | 0 | 1 | 2 | 3 | 4 | 5 | 6 | 7 | 8 | 9 | 10 | Very bad |
| --- | --- | --- | --- | --- | --- | --- | --- | --- | --- | --- | --- | --- |

1. **Emotional well-being**Considering your arthritis overall, how would you rate your level of emotional well-being during the past week? Circle the number that best describes your level of emotional well-being.

| Very good | 0 | 1 | 2 | 3 | 4 | 5 | 6 | 7 | 8 | 9 | 10 | Very bad |
| --- | --- | --- | --- | --- | --- | --- | --- | --- | --- | --- | --- | --- |

1. **Coping**Considering your arthritis overall, how well did you cope ( manage, deal, make do) with your disease during the last week ?

| Very well | 0 | 1 | 2 | 3 | 4 | 5 | 6 | 7 | 8 | 9 | 10 | Very poorly |
| --- | --- | --- | --- | --- | --- | --- | --- | --- | --- | --- | --- | --- |

**Calculation**RAID final value = (pain NRS value (range 0-10) x 0.21) + (function NRS value (range 0-10) x 0.16) + (fatigue NRS value (range 0-10) x 0.15) +  (phys well being NRS value (range 0-10) x 0.12) + (sleep NRS value (range 0-10) x 0.12) + (emotional well being NRS value (range 0-10) x 0.12) + (coping NRS value (range 0-10) x 0.12).

Thus, the range of the final RAID value is 0-10 where higher figures indicate worse status.

**Missing data imputation**If one of the 7 NRS values composing the RAID is missing, the imputation is as follows:

1. calculate the mean value of the 6 other (non-missing) NRS (range, 0-10)
2. impute this value for the missing NRS
3. Then, calculate the RAID as explained above. If 2 or more of the NRS are missing, the RAID is considered as missing value (no imputation)
