# Supplementary material for: Content validity of the Dutch Rheumatoid Arthritis Impact of Disease (RAID) score: results of focus group discussions in established rheumatoid arthritis patients and comparison with the International Classification of Functioning, Disability and Health core set for rheumatoid arthritis
Source: Arthritis Res Ther. 2016 Jan 22;18:22. doi: 10.1186/s13075-015-0911-z (PMC4722755; doi:10.1186/s13075-015-0911-z)
Supplement: Additional file 2: — WHO ICF score set for RA. The total WHO ICF core set for RA is shown. All categories are described in detail. (DOCX 21 kb) [file 13075_2015_911_MOESM2_ESM.docx]

**APPENDIX 2** WHO ICF core set for RA

| **ICF code** | **ICF category title** |
| --- | --- |
| **BODY FUNCTIONS** | |
| b130 | Energy and drive functions |
| b134 | Sleep functions |
| b152 | Emotional functions |
| b180 | Experience of self and time functions |
| b1801 | Body image |
| b280 | Sensation of pain |
| b2800 | Generalized pain |
| b2801 | Pain in body part |
| b28010 | Pain in head and neck |
| b28013 | Pain in back |
| b28014 | Pain in upper limb |
| b28015 | Pain in lower limb |
| b28016 | Pain in joints |
| b430 | Haematological system functions |
| b455 | Exercise tolerance functions |
| b510 | Ingestion functions |
| b640 | Sexual functions |
| b710 | Mobility of joint functions |
| b7102 | Mobility of joints generalized |
| b715 | Stability of joint functions |
| b730 | Muscle power functions |
| b740 | Muscle endurance functions |
| b770 | Gait pattern functions |
| b780 | Sensations related to muscles and movement functions |
| b7800 | Sensation of muscle stiffness |
| **BODY STRUCTURES** | |
| s299 | Eye, ear and related structures, unspecified |
| s710 | Structure of head and neck region |
| s720 | Structure of shoulder region |
| s730 | Structure of upper extremity |
| s73001 | Elbow joint |
| s73011 | Wrist joint |
| s7302 | Structure of hand |
| s299 | Eye, ear and related structures, unspecified |
| s710 | Structure of head and neck region |
| s720 | Structure of shoulder region |
| s730 | Structure of upper extremity |
| s73021 | Joints of hand and fingers |
| s73022 | Muscles of hand |
| s750 | Structure of lower extremity |
| s75001 | Hip joint |
| s75011 | Knee joint |
| s7502 | Structure of ankle and foot |
| s760 | Structure of trunk |
| s7600 | Structure of vertebral column |
| s76000 | Cervical vertebral column |
| s770 | Additional musculoskeletal structures related to movement |
| s810 | Structure of areas of skin |
| s73021 | Joints of hand and fingers |
| s73022 | Muscles of hand |
| s750 | Structure of lower extremity |
| **ACTIVITIES AND PARTICIPATION** | |
| d170 | Writing |
| d230 | Carrying out daily routine |
| d360 | Using communication devices and techniques |
| d410 | Changing basic body position |
| d415 | Maintaining a body position |
| d430 | Lifting and carrying objects |
| d440 | Fine hand use |
| d445 | Hand and arm use |
| d449 | Carrying, moving and handling objects, other specified and unspec. |
| d450 | Walking |
| d455 | Moving around |
| d460 | Moving around in different locations |
| d465 | Moving around using equipment |
| d470 | Using transportation |
| d475 | Driving |
| d510 | Washing oneself |
| d520 | Caring for body parts |
| d530 | Toileting |
| d540 | Dressing |
| d550 | Eating |
| d560 | Drinking |
| d570 | Looking after one’s health |
| d620 | Acquisition of goods and services |
| d630 | Preparing meals |
| d640 | Doing housework |
| d660 | Assisting others |
| d760 | Family relationships |
| d770 | Intimate relationships |
| d850 | Remunerative employment |
| d859 | Work and employment other specified and unspecified |
| d910 | Community life |
| d920 | Recreation and leisure |
| **ENVIRONMENTAL FACTORS** | |
| e110 | Products or substances for personal consumption |
| e115 | Products and technology for personal use in daily living |
| e120 | Products and technology for personal indoor and outdoor mobility and transportation |
| e125 | Products and technology for communication |
| e135 | Products and technology for employment |
| e150 | Design, construction and building products technology of buildings for public use |
| e155 | Design, construction and building products technology of buildings for private use |
| e225 | Climate |
| e310 | Immediate family |
| e320 | Friends |
| e340 | Personal care providers and personal assistants |
| e355 | Health professionals |
| e360 | Other professionals |
| e410 | Individual attitudes of immediate family members |
| e420 | Individual attitudes of friends |
| e425 | Individual attitudes of acquaintances, peers, colleagues, etc. |
| e450 | Individual attitudes of health professionals |
| e460 | Societal attitudes |
| e540 | Transportation services, systems and policies |
| e570 | Social security services, systems and policies |
| e580 | Health services, systems and policies |
